# Postpartum length of hospital stay among obstetric patients in Ibadan, Nigeria

**DOI:** 10.1186/s12913-024-11030-y

**Published:** 2024-05-04

**Authors:** Ikeola A. Adeoye, Blessing U. Aleka, Rotimi F. Afolabi, Timothy A.O Oluwasola

**Affiliations:** 1 Department of Epidemiology and Medical Statistics, Faculty of Public Health, College of Medicine, University of Ibadan, Ibadan, Nigeria; 2 Consortium of Advanced Research for Africa (CARTA), Nairobi, Kenya; 3 Department Obstetrics and Gynaecology, Faculty of Clinical Sciences, College of Medicine, University of Ibadan, Ibadan, Nigeria

**Keywords:** Length of hospital stay, Prolonged length of hospital stay, Vaginal delivery, Caesarean delivery, Complications

## Abstract

**Background:**

Postpartum Length of hospital stay (PLOHS) is an essential indicator of the quality of maternal and perinatal healthcare services. Identifying the factors associated with PLOHS will inform targeted interventions to reduce unnecessary hospitalisations and improve patient outcomes after childbirth. Therefore, we assessed the length of hospital stay after birth and the associated factors in Ibadan, Nigeria.

**Methods:**

We used the Ibadan Pregnancy Cohort Study (IbPCS) data, and examined the 1057 women who had information on PLOHS the mode of delivery [spontaneous vagina delivery (SVD) or caesarean section (C/S)]. The outcome variable was PLOHS, which was described as the time interval between the delivery of the infant and discharge from the health facility. PLOHS was prolonged if > 24 h for SVD and > 96 h for C/S, but normal if otherwise. Data were analysed using descriptive statistics, a chi-square test, and modified Poisson regression. The prevalence-risk ratio (PR) and 95% confidence interval (CI) are presented at the 5% significance level.

**Results:**

The mean maternal age was (30.0 ± 5.2) years. Overall, the mean PLOHS for the study population was 2.6 (95% CI: 2.4–2.7) days. The average PLOHS for women who had vaginal deliveries was 1.7 (95%CI: 1.5–1.9) days, whereas those who had caesarean deliveries had an average LOHS of 4.4 (95%CI: 4.1–4.6) days. About a third had prolonged PLOHS: SVD 229 (32.1%) and C/S 108 (31.5%). Factors associated with prolonged PLOHS with SVD, were high income (aPR = 1.77; CI: 1.13, 2.79), frequent ANC visits (> 4) (aPR = 2.26; CI: 1.32, 3.87), and antenatal admission: (aPR = 1.88; CI: 1.15, 3.07). For C/S: maternal age > 35 years (aPR = 1.59; CI: 1.02, 2.47) and hypertensive disease in pregnancy (aPR = 0.61 ; CI: 0.38, 0.99) were associated with prolonged PLOHS.

**Conclusion:**

The prolonged postpartum length of hospital stay was common among our study participants occurring in about a third of the women irrespective of the mode of delivery. Maternal income, advanced maternal age, ANC related issues were predisposing factors for prolonged LOHS. Further research is required to examine providers’ perspectives on PLOHS among obstetric patients in our setting.

## Background

Maternal and perinatal mortality remains a significant global concern despite receiving considerable attention as a worldwide public health priority over the past three decades in the form of the Safe Motherhood initiative [1], the fifth Millennium Development Goal (MDG 5) [2, 3] and the Sustainable Development Goal (SDG) 3.1 [4]. Unfortunately, maternal mortality remains high, with approximately 70% of these deaths occurring in sub-Saharan African countries, and Nigeria accounting for 28.5% of the global maternal deaths [5]. Direct preventable causes such as haemorrhage, anaemia, infection, hypertensive disorders of pregnancy, and obstructed labour account for more than 80% of maternal mortality in Nigeria [6]. Measures to reduce Maternal Mortality Ratio (MMR) include ensuring regular antenatal care, the presence of skilled healthcare providers at delivery, and promoting access to healthcare facilities equipped to provide emergency obstetric care. However, pregnant women may require hospitalisation for various reasons such as antenatal obstetric complications, high-risk pregnancies, and preterm labour.

Hospitalisation during pregnancy is a critical aspect of maternal and foetal health care. It is a period when a pregnant woman is admitted to the hospital for monitoring, treatment, and delivery of the baby. According to Ewing et al. (2011), approximately four million pregnant women aged 15–49 were hospitalised in the United States [7]. The quality of care provided during pregnancy and antenatal hospitalisation can significantly impact maternal and foetal health outcomes. There has been a notable increase in high-risk pregnancies due to maternal co-morbidities and complications with associated increase in the incidence of adverse maternal health outcomes and possible extension of the duration of postpartum hospital stay [8–10]. A study by Veras and Mathias (2014) in Panama, Brazil, showed that 37.8% of hospitalisations during the gestational period were due to obstetric complications [11]. Previous studies have also reported that hypertension, premature labour, foetal growth restriction, urinary tract infections (UTIs), and other complications are the leading causes of hospitalisation during pregnancy [12–14]. If these conditions are left untreated, they can result in unfavourable outcomes, including maternal, foetal, and infant mortality.

Length of hospital stay (LOHS) refers to the duration a woman spends in the hospital before, during, and after childbirth and is an essential measure of maternal care. According to the World Health Organization, the initial 24 h after childbirth represent a critical and high-risk period for mothers and their newborns [15]. Postpartum length of hospital stay (PLOHS) is an important indicator of the efficiency and quality of the maternal care the woman obtained [16–18]. A sufficient length of hospital stay after childbirth allows for a thorough assessment of both the mother and newborn, addressing any complications arising from childbirth, equipping mothers for exclusive breastfeeding and making allowance for adequate postnatal care [18]. Campbell et al. (2016) described the PLOHS across 92 low and middle-income countries and reported significant variations ranging from 1.3 to 6.6 days on the average, 0.5 and 6.2 days for singleton vaginal deliveries and 2.5 to 9.3 days for caesarean deliveries [19]. A short PLOHS may not allow enough time for the detection, diagnosis, and treatment of complications, potentially increasing morbidity and mortality risks. At the same time, prolonged PLOHS results in higher financial costs, increases the risk of hospital-acquired infections and causes sleep disturbances as well as breastfeeding difficulties, particularly in primigravidas [20–22]. For the past three decades, most developed countries have pushed for changes in their healthcare systems that will cut down on needless postpartum hospital stay [23]. These issues are yet to receive the much attention in low and middle income countries (LMICs) that bear a higher burden of maternal ill health.

Even though studies on PLOHS have emanated from Ghana [24], Ethiopia [25], Eriteria [18], and Sudan [26], these are lacking in Nigeria, despite bearing one of the highest burdens of maternal morbidity and mortality in sub-Saharan Africa. Investigating the PLOHS in Nigeria is crucial for assessing the quality of care obtained by obstetric patients and the efficiency of hospital management practices. This study will also provide evidence for targeted interventions to reduce unnecessary hospitalisations and improve patient outcomes. Therefore, we investigated PLOHS and the factors associated with prolonged PLOHS among women who had vaginal delivery and caesarean sections in Ibadan, Nigeria.

## Materials and methods

### Study design and sampling technique

**Fig. 1:**
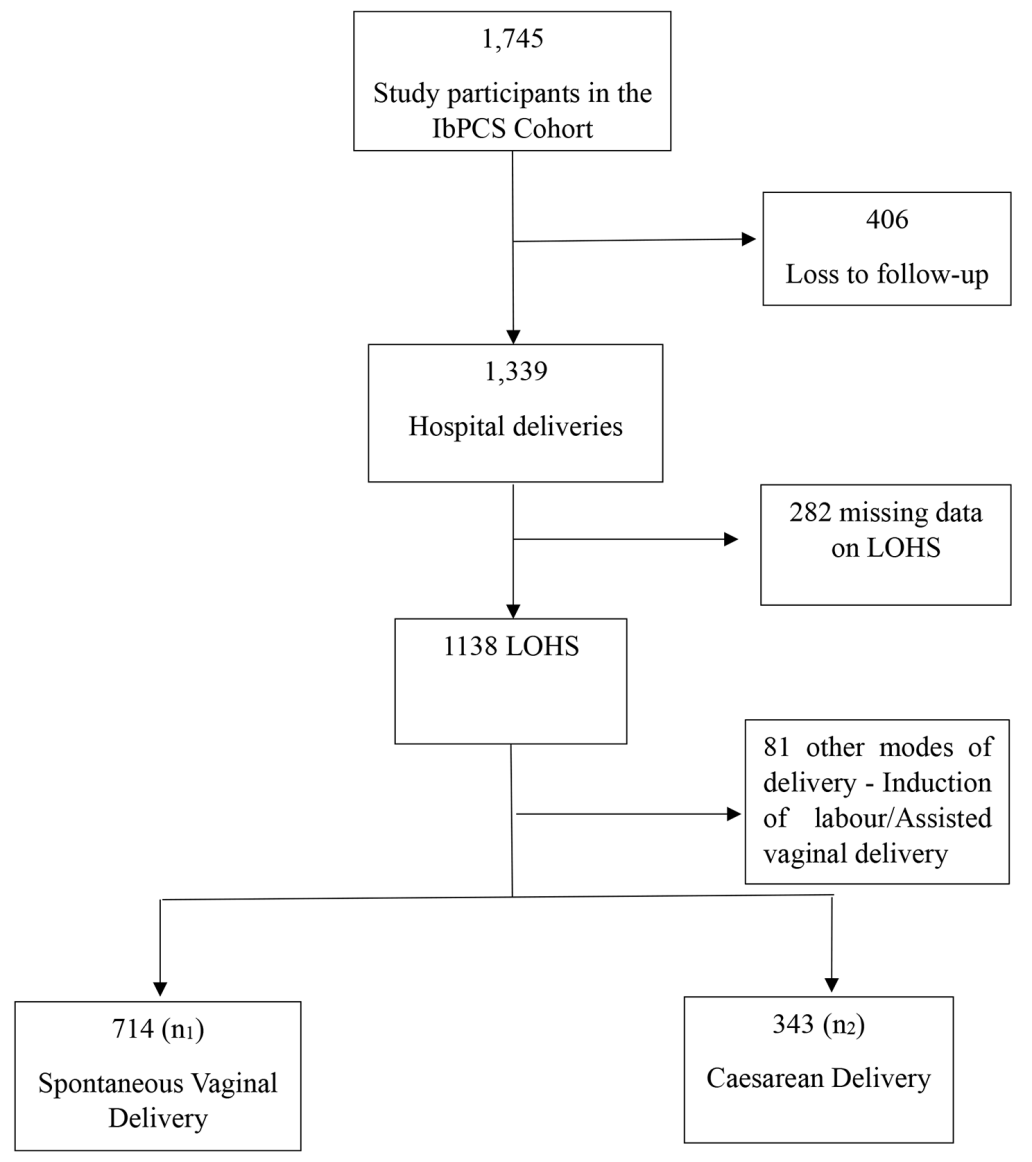
Flowchart of obstetric patients by PLOHS

The Ibadan Pregnancy Cohort Study (IbPCS) is a prospective cohort study on maternal obesity and lifestyle factors and their associated pregnancy and postpartum outcomes in Ibadan, Nigeria. IbPCS was conducted for eighteen months (between April 2018 and September 2019; the full description of the methodology has been documented [27]. In summary, 1,745 pregnant women with early gestational age (≤ 20 weeks) who met the eligibility criteria were enrolled on the study during their first antenatal booking visit and followed up until delivery from four facilities in Ibadan. These facilities were the University College Hospital, Adeoyo Maternity Teaching Hospital, Jericho Specialist Hospital, and Saint Mary Catholic Hospital, Oluyoro. The eligibility criteria were women at least 18 years of age and presented for antenatal care at an early gestational age (≤ 20 weeks) without severe medical conditions. Data were collected using pretested, interviewer-administered questionnaires and a desktop review of medical records. This current study is a secondary analysis of the IbPCS data, which were not lost to follow-up, delivered their babies at the facilities where they obtained their antenatal care and had a record of the delivery dates and discharge from the health facility. Women who met these selection criteria were examined in this study. A total of 1057 women who had data on LOHS and SVD (n_1_ = 714) or C/S (n_2_ = 343) were included in the current study (Fig. 1).

### Measures

The outcome variable was PLOHS, the time interval (measured in hours) between infant delivery and discharge from the health facility after childbirth. The postpartum LOHS was then categorised as normal PLOHS and prolonged PLOHS according to the mode of delivery (vaginal or caesarean). Postpartum LOHS was defined as normal if ≤ 24 h for vaginal delivery and ≤ 96 h for caesarean delivery but prolonged PLOHS if the cut-off is exceeded.

### Exposure variables

The following exposure variables were included: maternal age (in years), marital status (single or married), education (≤ primary, secondary, and tertiary), religion (Christianity and Islam), employment status (unemployed or employed), monthly income (< 20,000, 20,000–99,999 and > 100,000), ethnicity (Yoruba or non-Yoruba), parity (nullipara, 1–3 parity, and ≥ 4), ANC (< 4 visits and ≥ 4), Antenatal admission (yes/no), Body mass index (underweight, normal weight, overweight, obese), birth weight (low “< 2500 g”, normal “2500–3999 g”, macrosomia “≥ 4000 g”), Birth Asphyxia was defined as APGAR score < 7 at the one minute, postpartum haemorrhage (yes/no), gestational diabetes mellitus (yes/no), and perineal tear (yes/no).

Caesarean section (C/S) refers to the delivery of the baby through a surgical procedure on the abdomen [28]. Spontaneous vaginal delivery implies the delivery per vaginum (naturally without being induced). Perineal tears refer to lacerations in the perineum that occur during normal or instrumental vaginal delivery [28]. Low birth weight is defined as weight < 2.5 kg [28]. Postpartum haemorrhage refers to blood loss ≥ 500mls post-vaginal delivery and ≥ 1000mls post-caesarean section. Hypertensive disease of pregnancy was defined as elevated blood pressure – SBP ≥ 140 mmHg and DBP ≥ 90 mmHg or SBP ≥ 140 mmHg or DBP ≥ 90 mmHg. Gestational diabetes mellitus (GDM) is glucose intolerance first recognised during pregnancy. GDM diagnosis was made based on the International Association of Diabetic and Pregnancy Study Group (IADPSG) criteria in which GDM was present if one of the thresholds FPG ≥ 5.1 mmol/l; 1-hour plasma glucose ≥ 10.0 mmol/l, 1-hour plasma glucose ≥ 8.5 mmol/l was surpassed. GDM was diagnosed based on a 75-g 2-hour oral glucose tolerance test (OGTT) at pregnancy 24–28 weeks [29].

### Statistical analysis

Data were analysed using STATA (version 13.0 SE). Descriptive statistics, chi-square tests, and modified Poisson regression analyses were performed. Descriptive statistics are provided as mean (standard deviation) for continuous variables, while categorical variables are expressed as frequencies and percentages. We also used box plots and Kaplan-Meier graphs to assess the distribution of prolonged length of hospital stay. Chi-square tests evaluated the associations of the selected background characteristics with LOHS status by the women’s mode of delivery (vaginal delivery or caesarean section). A bivariate and multivariable modified Poisson regression model with robust error variance was used to identify the predictors of prolonged LOHS. Only variables with a *p*-value < 0.05 at bivariate levels using unadjusted models were included in the adjusted (final) model for prolonged PLOHS following vaginal delivery – income, religion, number of antenatal clinic (ANC) visits, antenatal admission, hypertensive disease of pregnancy and perineal tear were used in the final model. For prolonged PLOHS following C/S – maternal age, hypertensive disease of pregnancy, preterm delivery and LBW were used in the final model. The prevalence ratio (PR), 95% Confidence Interval (CI), and *p*-value were reported. All analyses were conducted at the 5% significance level.

## Results

### Characteristics of study participants

The baseline characteristics of the study participants are shown in Table 1. The mean age and mean gestational age at delivery were 30.0 ± 5.2 years and 38.7 ± 1.9 weeks, respectively. The majority were married 1,000 (94.6%) and employed 933 (88.3%). The association between the PLOHS and women’s characteristics by mode of delivery are presented in Table 2. A total of 1057 women were investigated for their postpartum length of hospital stay, of which 714 (67.5%) had vaginal delivery and 343(32.5%) had caesarean section, respectively. About a third of the participants had prolonged PLOHS: vaginal delivery, 229 (32.1%), and caesarean section 108 (31.5%). Among women with vaginal delivery, the proportion with prolonged PLOHS differed significantly by maternal education (*p* < 0.001), income (*p* < 0.001), ANC visits (*p* < 0.001), and ANC admission (*p* = 0.002). Conversely, among the women who had caesarean delivery, those aged ≥ 35 years experienced PLOHS more than younger women (*p* = 0.002).

### Length of hospital stay

**Fig. 2:**
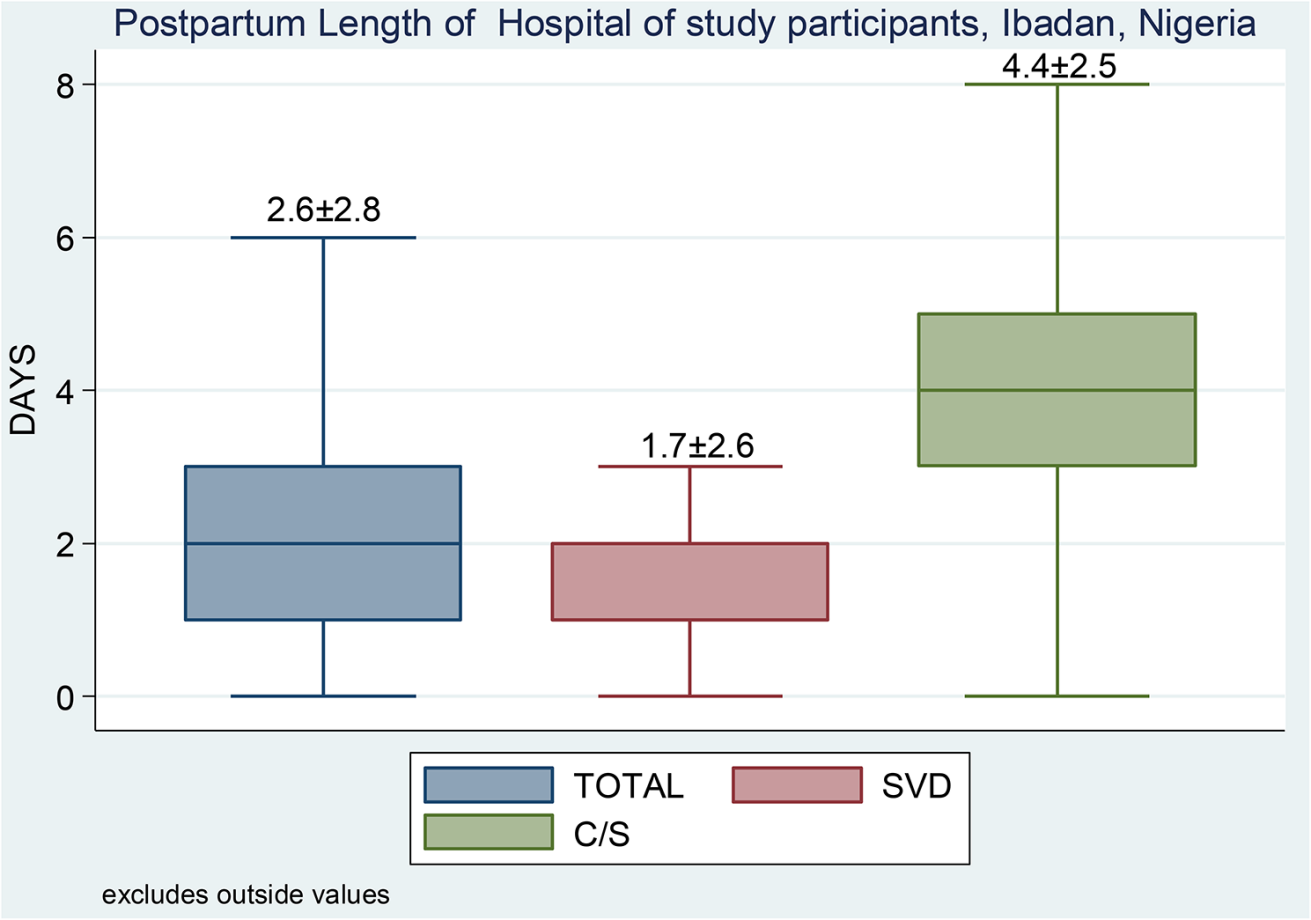
Boxplot of the postpartum length of hospital stay of study participants

**Fig. 3:**
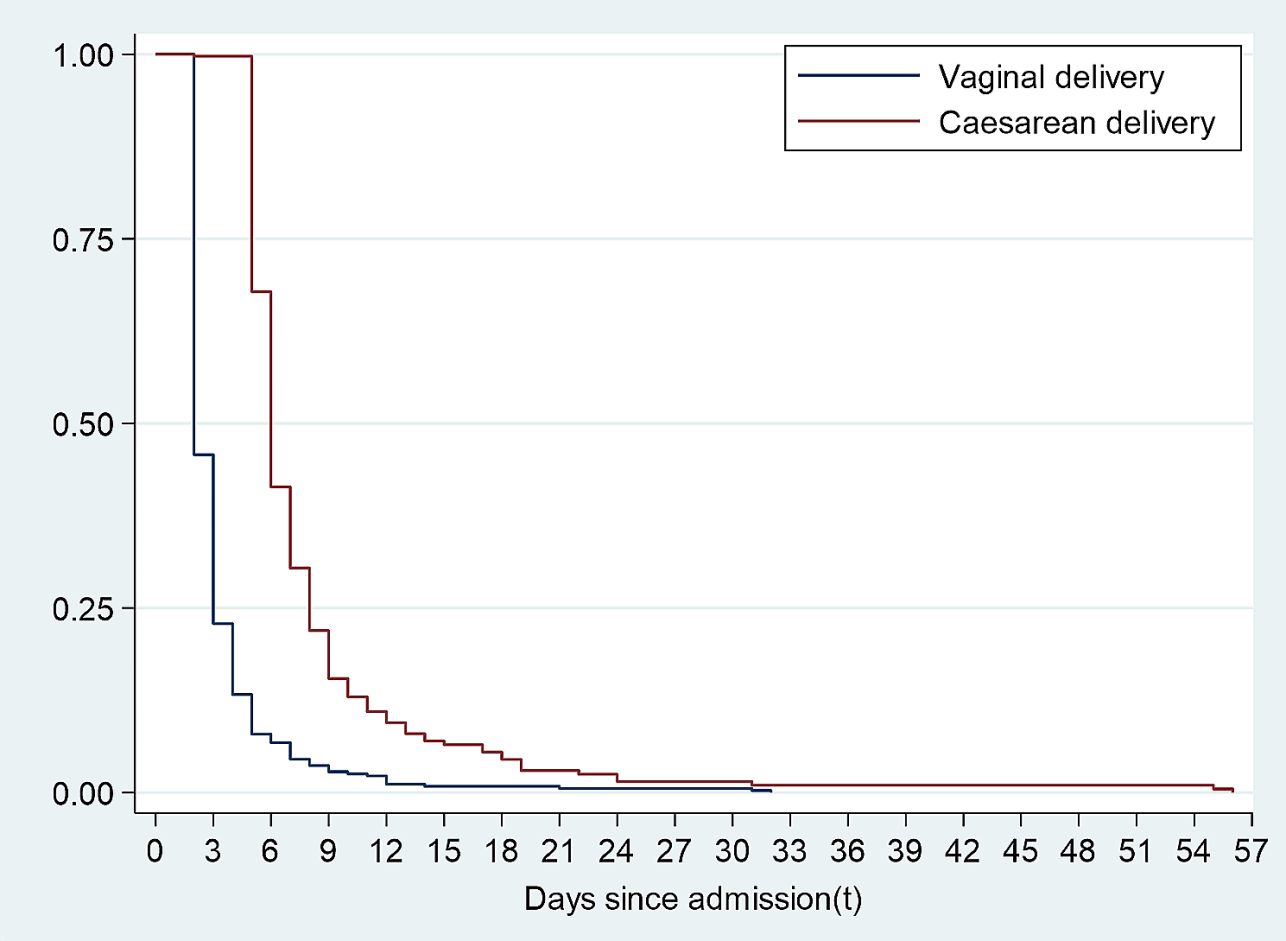
Overall survival curve showing the probability of having prolonged LOHS by mode of delivery

Figure 2 shows the postpartum length of hospital stay for the study participants. Overall, the mean PLOHS for the study population was 2.6 ± 2.8 days (61.7 ± 68.5) hours. The average PLOHS for women who had vaginal deliveries was 1.7 ± 2.6 days (41.0 ± 62.2) hours, whereas those who underwent caesarean deliveries had an average LOHS of 4.4 ± 2.5 days (104.1 ± 59.7) hours. Figure 3 shows the probability of prolonged PLOHS by the mode of delivery. On average, the risk of having prolonged PLOHS was higher among women with a caesarean section than those with a vaginal delivery.

### Factors associated with prolonged hospital stay

The factors associated with postpartum LOHS among women who delivered vaginally are presented in Table 3. The unadjusted and adjusted prevalence ratios (aPR) and 95% CI of prolonged LOHS for women with vaginal delivery were presented using the modified Poisson regression model with robust variance. The significant factors included maternal income ([PR = 1.74; CI: 1.34, 2.25], ANC visits (PR = 1.09; CI: 1.25, 2.88), ANC admission (PR = 1.61; CI: 1.11, 2.34), Hypertensive disease of pregnancy (PR = 0.69; CI: 0.43, 0.99), perineal tear (PR = 1.76; CI: 1.35, 2.31). However, when subjected to multivariate analysis, only higher income ([aPR = 1.77; CI: 1.13, 2.79], ANC visits (aPR = 2.26; CI: 1.32, 3.87) and antenatal admission (aPR = 1.88; CI: 1.15, 3.07) remained significantly associated with prolonged PLOHS.

The factors associated with a prolonged PLOHS in women who undertook caesarean section are shown in Table 4. Women ≥ 35 years (PR = 1.60; CI: 1.08, 2.36), hypertensive disease of pregnancy (PR = 0.63; CI:0.40, 0.99), Women with preterm babies (PR = 1.64; CI:1.05, 2.37), low birth weight babies (PR = 2.00; CI: 1.23, 3.27) had a higher likelihood of prolonged PLOHS than those with normal-weight babies. After adjusting for variables in the final model, women ≥ 35 years (aPR = 1.59; CI: 1.02, 2.47) and hypertensive disease of pregnancy (aPR = 0.61; CI: 0.38, 0.99) remained significantly associated with prolonged LOHS.

**Table 1:** Baseline characteristics of the study participants, Ibadan, Nigeria

Characteristics	*N* 1,057 (frequency)	Percentage (*n*/*N*)
**Age group**		
< 20	16	1.5
20–29	494	46.7
30–39	503	47.6
≥ 40 years	44	4.2
**Mean age (years)**	**30.0 (± 5.2)**	
**Parity**		
Nulliparous	470	43.8
2–4	535	51.0
≥ 5	45	4.3
**Marital Status**		
Single	57	5.4
Married	1,000	94.6
**Level of Education**		
≤ Primary	22	2.1
Secondary	272	25.8
Tertiary	761	72.1
**Employment status**		
Employed	933	88.3
Unemployed	124	11.7
**Religion**		
Christianity	623	59.2
Islam	429	40.8
**Ethnicity**		
Yorubas	940	89.2
Non-Yorubas	114	10.8
**Income per month (Naira)**		
< 20,000	329	35.5
20,000–99,999	532	57.5
≥ 100,000	65	7.0

**Table 2:** Association between LOHS and participants’ characteristics by mode of delivery

Characteristics	Vaginal Delivery			Caesarean Section			
	Normal LOH *n*(%)	Prolonged LOH *n* (%)	*p*-value	Normal LOH *n*(%)		Prolonged LOH *n*(%)	*p*-value
**Overall**	485 (67.9)	**229 (32.1)**		235 (68.5)		**108 (31.5)**	
**Age (Years)**							
< 35	406 (69.0)	187 (31.5)	0.495	181 (73.0)		67 (27.0)	**0.004**
≥ 35	79 (65.3)	42 (34.7)		54 (56.8 )		41 (43.2)	
**Mean Age (SD)**	29.0 ± 5.1	30.0 ± 4.8	**0.007**	30.9 ± 5.1		32.7 ± 5.4	**0.004**
**Marital Status**							
Single	33(78.57)	9(21.43)		12 (80.0)		3(20.0)	
Married	452 (67.26)	220(32.74)	0.128	223 (68.0)		105(32.0)	0.327
**Education**							
Primary	23 (66.7)	6 (33.3)		3 (75.0)		1(25.0)	
Secondary	168 (80.0)	42 (20.0)		41 (66.1)		21 (33.9)	
Tertiary	305 (62.8)	181 (37.2)	**0.000**	189(68.7)		86(31.3)	0.886
**Employment**							
Unemployed	66 (71.0)	27 (29.0)		22 (71.0)		9(29.0)	
Employed	419 (67.5)	202 (32.5)	0.501	213 (68.5)		108 (31.5)	0.758
**Religion**							
Christianity	235 (61.0)	150 (9.0)		157 (66.8)		79 (33.2)	
Islam	247 (76.0)	78 (24.0)	**0.000**	75 (72.1)		29 (27.9)	0.331
**Ethnicity**							
Non-Yorubas	39 (57.4)	29 (42.7)		33 (71.7)		13 (28.3)	
Yorubas	445 (69.2)	198 (30.8)	0.046	202 (68.0)		95 (32.0)	0.613
**Income (**₦)							
< 20,000	204 (81.0)	48 (19.1)		55 (71.4)		77 (100.0)	
20,000–99,999	195 (59.5)	133(40.6)		138 (67.7)		204 (100.0	
> 100,000	19 (54.3)	16 (45.7)	**0.000**	21 (70.0)		9(30.0)	0.821
**Parity**							
Nullipara	206 (65.6)	108 (34.4)		113 (72.5)		43 (27.6)	
1–3	254(69.8)	110 (30.2)		115 (67.3)		56 (32.8)	
4 and above	23 (71.9)	9 (28.1)	0.454	7(46.2)		7(53.9)	0.119
**BMI**							
Underweight	14 (73.7)	5 (26.3)		3(60.0)		2 (40.0)	
Normal	267(70.5)	112 (30.0)		83(68.6)		38 (31.4)	
Overweight	123 (63.1)	72 (37.0)		75(66.4)		38 (33.6)	
Obese	71 (68.3)	33 (31.9)	0.320	65(68.4)		30 (31.6)	0.961
**Antenatal Visits**							
< 4 visits	94(77.7)	27 (22.3)		41 (78.9)		11 (21.2)	
4 or more visits	172 (57.3)	127 (42.5)	**0.000**	118 (67.1)		58 (33.1)	0.104
**Antenatal Admission**							
No	419 (69.5)	184 (30.5)		180 (70.9)		74 (29.1)	
Yes	34 (50.8)	33 (49.2)	**0.002**	44 (62.5)		27 (37.5)	0.175;
**Hypertensive Disease of Pregnancy**							
No	361 (65.9)	187 (34.1)		149 (64.5)		82 (35.5)	
Yes	117 (76.5)	36 (23.5)	**0.013**	83 (77.6)		24 (22.4)	**0.016**
**Gestational Diabetes Mellitus**							
No	193 (70.2)	82 (29.8)		94 (73.4)		30 (26.1)	
Yes	44 (67.7)	21 (32.3)	0.694	18 (60.0)		12 (40.0)	0.145
**Postpartum Haemorrhage**							
No	450 (68.4)	208 (31.6)		164 (67.2)		80 (25.5)	
Yes	35 (65.5)	21 (37.5)	0.365	71 (71.7)		99 (45.0)	0.416
**Perineal Tear**							
No	342 (74.5)	117 (25.5)		200 (69.4)		88 (30.6)	
Yes	121 (55.0)	99 (45.0)	**0.000**	2 (66.7)		1 (33.3)	0.917

Frequency (n), percentage (%), Mean (M) ± standard deviation (SD)

**Table 3:** Factors associated with prolonged length of hospital stay among women with vaginal deliveries

Characteristics	Unadjusted	*p*-value	Adjusted	*p*-value
	Prevalence Ratio (PR) (95% CI)		Prevalence Ratio (PR) (95% CI)	
**Age (Years)**				
< 35	1			
> 35	1.10 (0.79–1.54)	0.574		
**Marital Status**				
Single	1			
Married	1.53(0.78– 2.96)	0.213		
**Education**				
Primary	1			
Secondary	0.60 (0.26–1.41)	0.242		
Tertiary	1.12(0.50–2.52)	0.789		
**Employment**				
Unemployed	1			
Employed	1.12 (0.75–1.67)	0.579		
**Religion**				
Christianity	1		1	
Islam	0.61 (0.47–0.81)	**0.001**	0.71 (0.47–1.06)	0.098
**Ethnicity**				
Non-Yorubas	1			
Yorubas	0.72 (0.49–1.07)	0.101		
**Income (**₦)				
< 20,000	1		1	
20,000–99,999	1.74(1.34–2.25)	0.001*	1.77(1.13–2.79)	0.013*
> 100,000	1.97(1.24–3.13)	0.004*	1.70 (0.82–3.56)	0.074
**Obstetric Characteristics**				
**Parity**				
Nullipara	1			
1–3	0.88 (0.67–1.55)	0.339		
≥ 4	0.82 (0.41–0.95)	0.562		
**BMI**				
Underweight	1			
Normal	1.22(0.46–2.75)	0.800		
Overweight	1.40 (0.57 − 3.47)	0.464		
Obese	1.21 (0.47 − 3.09)	0.697		
**Antenatal Visits**				
< 4 visits	1		1	
4 or more visits	1.09 (1.25 − 2.88)	0.002*	2.26 (1.32 − 3.87)	0.003*
**Antenatal Admission**				
No	1		1	
Yes	1.61 (1.11 − 2.34)	**0.011**	1.88 (1.15 − 3.07)	**0.011**
**Hypertensive Disease of Pregnancy**				
No	1		1	
Yes	0.69 (0.48–0.99)	**0.041**	0.78 (0.47–1.29)	0.341
**Gestational Diabetes Mellitus**				
No	1			
Yes	1.08 (0.67– 1.75)	0.743		
**Postpartum Haemorrhage**				
No	1			
Yes	1.19(0.76 − 1.86)	0.456		
**Perineal Tear**				
No	1		1	
Yes	1.76 (1.35 2.31)	**< 0.001**	1.36 (0.93–1.98)	0.105
**Perinatal Factors**				
**Preterm**				
No	1			
Yes	0.97(0.65–1.43)	0.865		
**Apgar Score at 1 min**				
No	1			
Yes	1.05 (0.65–1.70)	0.830		
**Birth Weight**				
Normal birth weight	1			
Low birth weight	1.15 (0.68–1.95)	0.592		
Macrosomia	0.97(0.37–1.26)	0.937		

**Table 4:** Factors associated with prolonged length of hospital stay among women with Caesarean deliveries

Characteristics	Unadjusted	*p*-value	Adjusted	*p*-value
	Prevalence Ratio (PR) (95% CI)		Prevalence Ratio (PR) (95% CI)	
**Age (years)**				
< 35	1		1	
≥ 35	1.60 (1.08 − 2.36)	0.018*	1.59 (1.02 − 2.47)	0.039*
**Marital Status**				
Single	1			
Married	1.60(0.51–5.04)	0.422		
**Education**				
Primary	1			
Secondary	1.35(0.18–10.07)	0.767		
Tertiary	1.25 (0.17–8.98)	0.824		
**Employment Status**				
Unemployed	1			
Employed	1.09(0.55– 2.16)	0.799		
**Religion**				
Christianity	1			
Islam	0.84 (0.55–1.29)	0.422		
**Ethnicity**				
Non-Yorubas	1			
Yorubas	1.13 (0.63–2.02)	0.675		
**Income (**₦)				
< 20,000	1			
20,000–99,999	1.13 (0.70–1.61)	0.570		
> 100,000	1.15(0.65–2.03)	0.636		
**Parity**				
Nullipara	1			
1–3	1.19 (0.80–1.83)	0.614		
≥ 4	1.95 (0.88 − 4.34)	0.902		
**BMI**				
Underweight	1			
Normal	0.79(0.19–3.25)	0.831		
Overweight	0.84 (0.20–3.48)	0.811		
Obese	0.79 (0.19–3.30)	0.746		
**Antenatal Visits**				
< 4 visits	1			
4 or more visits	1.56 (0.82–2.97)	0.178		
**Antenatal Admission**				
No	1			
Yes	1.28 (0.82 − 2.00)	0.262		
**Hypertension Disease of Pregnancy**				
No	1		1	
Yes	0.63 (0.40–0.99)	**0.048**	0.61 (0.38–0.99)	0.048*
**Gestational Diabetes Mellitus**				
No	1			
Yes	1.05 (0.78–2.91)	0.223		
**Postpartum Haemorrhage**				
No	1			
Yes	0.86 (0.56–1.34)	0.501		
**Perineal Tear**				
No	1			
Yes	1.09 (0.15–7.83)	0.931		
**Perinatal Factors**				
**Preterm**				
No	1		1	
Yes	1.64 (1.05–2.57)	0.029*	1.39 (0.82–2.35)	0.215
**Apgar Score at 1 min**				
No	1			
Yes	1.16 (0.68–1.96)	0.589		
**Birth Weight**				
Normal birth weight	1		1	
Low birth weight	2.00 (1.23–3.27)	0.005*	1.63 (0.91–2.91)	0.097
Macrosomia	1.43 (0.74–2.77)	0.291	1.24 (0.61–2.51)	0.556

## Discussion

Postpartum length of hospital stay is an important indicator of the efficiency and quality of hospital-based delivery care. Postpartum LOHS has been extensively reported in developed countries [30–34], with greater emphasis on short PLOHS because of the need to prevent nosocomial infections, patient dissatisfaction and higher hospital costs [17, 19, 25], even though it’s associated with a higher risk of hospital readmissions [33]. Conversely, prolonged PLOHS, which results from pregnancy complications, is often of more significant concern in LMIC because of the risks of nosocomial infections, maternal sleeping disorders, breastfeeding difficulties, and increased maternal stress [15, 25, 26]. Studies on postpartum LOHS are lacking in Nigeria despite evidence emerging from some African countries [18, 24–26]. Understanding PLOHS among obstetric patients will foster the efficient healthcare resource allocation, such as adequate staffing and bed allocation, and facilitate performance comparisons with other facilities and settings to identify best practices and areas for improvement [35, 36]. Therefore, using the Ibadan Pregnancy Cohort Study, we assessed PLOHS and examined the factors associated with prolonged PLOHS among obstetric patients in Ibadan. In this study, the average LOHS was 2.6 days, similar to the study in Eastern Sudan, which reported an average LOHS of 2.7 days [26]. But lower compared to reports from India (3.4 days) [37] and Australia (4 days) [38]. Notably, more developed countries in the United States, Canada, and the United Kingdom have documented a shorter length of less than two days [33, 34].

Obstetric PLOHS has been found to vary by mode of delivery, and researchers have reported PLOHS for vaginal delivery and caesarean section [16, 17, 19, 26, 32, 37]. Expectedly, the LOHS for caesarean sections is longer than vaginal deliveries because these women require a longer time to recover. They also have a higher risk of morbidity [26]. In this study, vaginal delivery PLOHS and caesarean section LOHS were 1.7 days and 4.4 days, respectively. Whereas India, which faces similar maternal health challenges as Nigeria, both countries being the major contributors to the burden of maternal morbidity and mortality globally [5], reported comparable vaginal delivery PLOHS (2.1 days) as found in our study [37] but a much higher caesarean section LOHS (8.6 days) [37]. Campbell and colleagues (2016) estimated PLOHS from 92 countries, including 30 LMICs. They reported an overall PLOHS: of 1.3–6.6 days, vaginal delivery PLOHS 0.5–6.2 days and 2.5–9.3 days for caesarean Sect [19]. Other countries that have reported PLOHS for vaginal delivery and caesarean section are Australia (4 days; 6.2 days) [39], North Eastern Italy (4 days; 6.2 days) [31, 32] Nepal (4 days; 7 days) [40]. In sub–Saharan Africa, a recent study by Tsiga Ahmed et al. in 2022 reported the LOHS after childbirth across three African countries (South Africa, Ghana, and Malawi) [41]. While vaginal PLOHS were similar (1.5 days), they observed variations for caesarean section PLOHS according to the level of the health care system across the three countries: South Africa (3.5 days), Ghana (4.5 days), and Malawi (7.5 days) [41].

In this study, prolonged PLOHS occurred in a third of our study population: vaginal delivery (32.1%) and caesarean Sect. (31.5%). Among women who had vaginal delivery, the factors significantly associated with prolonged LOHS included maternal income, religion, ANC visits, ANC admission and perineal tear. However, in the final model, only maternal income, ANC visits and ANC admission remained significant. Women with higher income had about a twofold likelihood of prolonged LOHS than low-income earners. The Indian study also corroborated this observation [37]. Plausibly, financially stable women have additional resources and higher purchasing power to procure better, higher-quality care, which may require a longer time in the hospital. Also, they are likely to have a higher knowledge about pregnancy complications and a higher willingness to pay for services than women with lower earning power.

On the other hand, other studies have reported that wealthier women are less likely to have prolonged PLOHS because of higher autonomy and self-efficacy as well as appropriate antenatal care culminating in adequate birth preparedness and complication readiness [19, 25]. We also noted that Muslim women were less likely to have prolonged PLOHS. This finding has been confirmed by other researchers [37, 42]. This association may be related to certain religious/cultural practices associated with naming the infant. However, the association became insignificant after adjusting for confounders.

We observed that women with frequent ANC visits had had higher odds for prolonged PLOHS among those who had SVD. The Indian study also reported that women with four or more ANC visits in their population were more likely to have a prolonged stay [37]. The initial indications for frequent antenatal visits may be unresolved till delivery thus necessitating a prolonged postpartum LOHS. For similar reasons, women who had ANC admission also had a greater likelihood of prolonged PLOHS. Among our study participants, the common reasons for antenatal admission included hyperemesis gravidarium, Hypertensive Disorders of Pregnancy, and premature contractions. Surprisingly, having HDP was protective of prolonged PLOHS among women who had SVD or C/S. These can be attributed to the adequate control of their blood pressure, appropriate case selection and advice on mode of delivery as well as closer monitoring of these patients both antenatally and in labour since all the study participants were booked patients and obtained care from comprehensive obstetric care facilities.

Maternal and perinatal complications are crucial reasons for protracted hospital stay. We found that women with perineal tears were more likely to have a prolonged hospital stay than those without tears. The reasons for an extended stay among women with perineal tears include surgical repair, close monitoring, pain management, and infection control with parenteral antibiotics [28]. However, the relationship became insignificant in the final model after adjusting for confounders. Although previous studies had reported the association between maternal obesity and prolonged PLOHS because of the associated pregnancy complication, no such association was found in this study [38, 43].

Among women who had caesarean section, advanced maternal age (≥ 35 years), hypertensive disease in pregnancy, preterm and low birth weight (LBW) babies were the factors associated with prolonged LOHS on bivariate analysis. However, in the final model, only higher maternal age and hypertensive disease in pregnancy were retained. The association between older maternal age and LOHS has been documented in the literature [19, 37, 44]. Older maternal age could influence the PLOHS cause of the higher risk of obstetric complications and co-morbidities, which require treatment and a more extended stay in the hospital. Also, it has been suggested that older women are likely to have more knowledge about pregnancy care and have higher autonomy in decision-making in maternal care utilisation [44].

Neonatal complications are independent risk factors for prolonged PLOHS because of the need for treatment and hospital care. We observed in this study that mothers of LBW infants and premature births had higher odds for PLOHS especially when delivered via C/S. This finding has been confirmed by several researchers across different settings [16, 19, 41, 45]. LBW from premature births or intrauterine growth restriction often have various challenges, including respiratory difficulties from immature lungs, feeding difficulties, and poor temperature regulation. Therefore, LBW babies may require specialised care, including neonatal intensive care, oxygen supplementation, incubator care, and nasogastric tube feeding, leading to an extended LOHS for their mothers [25, 31, 32]. Although LBW and preterm delivery became statistically insignificant at multivariate analysis, this should be interpreted with caution in view of the clinical implication, and public health importance, of the finding on those babies.

Our study has several strengths. First, it fills an important gap in maternal and perinatal epidemiology in Nigeria - postpartum LOHS - among a large cohort of obstetric patients. We also examined a broad range of variables, including demographic, clinical, and obstetric characteristics, which enabled a comprehensive analysis of factors associated with prolonged LOHS in Nigeria. Unlike other studies that estimated their LOHS using secondary data or self-reporting, which is associated with recall bias, uncertainty, and reporting bias, this study objectively assessed LOHS by direct ascertainment from medical records and discharge summaries. Nonetheless, this study does have certain limitations, including the loss of follow-up during the Ibadan pregnancy cohort study (IbPCS). Also, women who did not have hospital deliveries did not have a record of their LOHS, and there were limitations in the generalizability of findings to women who obtained care in private healthcare facilities, primary healthcare centres, or rural areas. Postpartum anaemia and other postoperative complications, such as wound infections, could not be analysed in the study and, hence, should be considered in future studies as well as the providers’ perspectives on LOHS among obstetric patients.

## Conclusion

This research investigated the postpartum LOHS among obstetric patients in Ibadan, Nigeria. The average PLOHS after childbirth was 2.7 days, 1.7 days for SVD and 4.4 days for C/S. Factors associated with prolonged PLOHS included older maternal age, high income, religion, frequent ANC, ANC admission, and Hypertensive disease of pregnancy. Therefore, healthcare providers and policymakers should provide targeted interventions, which include improved protocols for managing high-risk cases and enhancing postpartum care to reduce the length of hospital stays for obstetric patients, leading to improved patient experiences, more efficient resource utilisation, and enhanced postpartum recovery.

## Data Availability

The datasets generated and analysed during the current study are not publicly available because they contain potentially identifying and confidential information but are available from the corresponding author on reasonable request if they meet the criteria for accessing confidential data.
